# Revealing the regulation of allergic asthma airway epithelial cell inflammation by STEAP4 targeting MIF through machine learning algorithms and single-cell sequencing analysis

**DOI:** 10.3389/fmolb.2024.1427352

**Published:** 2024-08-08

**Authors:** Lu Qiao, Shi-meng Li, Jun-nian Liu, Hong-lei Duan, Xiao-feng Jiang

**Affiliations:** ^1^ Department of Clinical Laboratory, The Fourth Affiliated Hospital of Harbin Medical University, Harbin, Heilongjiang, China; ^2^ Department of Clinical Laboratory, China-Japan Union Hospital of Jilin University, Changchun, Jilin, China; ^3^ Department of Digestive, Weihai Municipal Hospital, Weihai, Shandong, China

**Keywords:** allergic asthma, airway epithelial cells, machine learning algorithms, STEAP4, MIF

## Abstract

Asthma comprises one of the most common chronic inflammatory conditions, yet still lacks effective diagnostic markers and treatment targets. To gain deeper insights, we comprehensively analyzed microarray datasets of airway epithelial samples from asthmatic patients and healthy subjects in the Gene Expression Omnibus database using three machine learning algorithms. Our investigation identified a pivotal gene, *STEAP4*. The expression of *STEAP4* in patients with allergic asthma was found to be reduced. Furthermore, it was found to negatively correlate with the severity of the disease and was subsequently validated in asthmatic mice in this study. A ROC analysis of *STEAP4* showed the AUC value was greater than 0.75. Functional enrichment analysis of *STEAP4* indicated a strong correlation with IL-17, steroid hormone biosynthesis, and ferroptosis signaling pathways. Subsequently, intercellular communication analysis was performed using single-cell RNA sequencing data obtained from airway epithelial cells. The results revealed that samples exhibiting low levels of *STEAP4* expression had a richer *MIF* signaling pathway in comparison to samples with high *STEAP4* expression. Through both *in vitro* and *in vivo* experiments, we further confirmed the overexpression of STEAP4 in airway epithelial cells resulted in decreased expression of MIF, which in turn caused a decrease in the levels of the cytokines IL-33, IL-25, and IL-4; In contrast, when the STEAP4 was suppressed in airway epithelial cells, there was an upregulation of MIF expression, resulting in elevated levels of the cytokines IL-33, IL-25, and IL-4. These findings suggest that STEAP4 in the airway epithelium reduces allergic asthma Th2-type inflammatory reactions by inhibiting the MIF signaling pathway.

## 1 Introduction

Asthma is a common, long-lasting inflammatory disorder affecting the air passages (Mims, 2015). The condition is defined by recurrent episodes of wheezing, expectorating sputum, reversible restriction of airflow, and heightened sensitivity of the airways to environmental bronchospasmic stimuli (such as dust mites, pollen, animal dander, and air pollution) (Holgate et al., 2015). Allergic asthma comprises the most common asthmatic phenotype. About 334 million individuals worldwide currently suffer from asthma, and around 250,000 deaths attributed to this condition each year, thus highlighting its significant impact on public health (Croisant, 2014). Despite the standardization of stepwise treatment for asthma, there are still patients who experience inadequate or only partial improvement of symptoms after receiving intensive treatment (Abu-Kishk et al., 2016). Furthermore, due to the limitations of conventional diagnostic techniques, a significant number of patients fail to receive a timely diagnosis and appropriate treatment, resulting in irreversible impairment to the functionality and structure of the lungs (Aaron et al., 2018). Thus, in light of the increasing prominence of precision medicine, it is imperative to understand the molecular pathways responsible for asthma and identify suitable biomarkers to accurately classify and address the condition (Kaur and Chupp, 2019).

Airway epithelial cells (AECs) serve as a physical, chemical, and immunological barrier between the submucosal layer of the airways and the external environment. They typically function as the initial defense mechanism, protecting the host from any potential harm caused by inhaled environmental particles (Hellings and Steelant, 2020). However, asthmatic patients display both structural and functional abnormalities in their AECs (Moheimani et al., 2016). The primary role of AECs is to maintain the integrity of the mucosal barrier and aid in the elimination of inhaled particles through mucociliary clearance (Lambrecht and Hammad, 2012). Asthmatic patients often experience damage to their AECs, which makes it easier for external irritants to reach the submucosal layer and come into contact with dendritic cells (DCs). This contact then triggers Th2 inflammatory responses (Loxham et al., 2014; Hellings and Steelant, 2020). In addition, AECs express a diverse range of pattern recognition receptors (PRRs) that enable them to rapidly identify pathogen-associated molecular patterns (PAMPs) from inhaled microorganisms, parasites, and allergens. They can also identify damage-associated molecular patterns (DAMPs) that are released when cells are damaged, die, or experience stress (Amarante-Mendes et al., 2018; Heijink et al., 2020). This stimulates the secretion of alarmin cytokines and chemokines from AECs, resulting in the infiltration of immune cells in the affected area (Yang et al., 2021). Extended exposure of AECs to PAMPs and DAMPs reduces the activation threshold of PRRs to stimuli, leading to exaggerated immune responses and facilitating the onset of asthma (Monick et al., 2003; Pace et al., 2008; Pouwels et al., 2014). Furthermore, in cases where the AECs are damaged, healthy individuals demonstrate a strong capacity for epithelial repair, rapidly restoring the integrity of the barrier (Iosifidis et al., 2016). In contrast, asthmatic patients exhibit an increased ability for cellular proliferation but have dysregulated differentiation, predominantly resulting in the formation of goblet cells (Inoue et al., 2019; Raby et al., 2023). The absence of ciliated cells further reduces the airway’s ability to resist the invasion of allergens. As observed, AECs play a crucial role in the development, progression, and acute exacerbation of asthma. Thus, future therapeutic strategies should prioritize the investigation and intervention of AECs’ functionality.

Machine learning algorithms comprise advanced mathematical models that enable computers to autonomously acquire knowledge, make decisions, and offer predictive outcomes from large-scale data (Binson et al., 2024). These algorithms have the potential to significantly enhance the effectiveness and accuracy of data processing, as well as reveal intricate patterns and associations that are concealed within complex datasets. Accordingly, the present study utilizes three advanced machine learning techniques to effectively identify *six-transmembrane epithelial antigen of prostate 4* (*STEAP4*), a crucial gene associated with asthma, from AEC microarray datasets. Additionally, single-cell RNA sequencing (scRNA-seq) data was used to further investigate the potential downstream signaling pathways of *STEAP4* in AECs. Finally, the accuracy of the predictions was confirmed from the results of cell culture and animal studies. The findings obtained from the study offer novel insights into the pathogenesis of asthma and is likely to facilitate the development of future therapeutic strategies.

## 2 Materials and methods

### 2.1 Data preprocessing

The Gene Expression Omnibus (GEO) database was searched to retrieve the original microarray datasets GSE41861, GSE4302, GSE43696, and GSE63142. Table 1 displays the attributes of the datasets. Additionally, in the GSE43696 dataset, 88 asthma patients are divided into 50 mild-moderate asthmatic and 38 severe asthmatic. In the GSE63142 dataset, 128 asthma patients are divided into 72 mild-moderate asthmatic and 56 severe asthmatic. The assessment of asthma severity has been extensively discussed in previous studies (Reddel et al., 2009). The expression profiles of the GSE41861 and GSE4302 datasets were combined to create a discovery set, while the GSE43696 and GSE63142 datasets were used to form a test set. The removal of the batch effect was carried out using the “ComBat” function from the “sva” package in R (version 4.1.2). A principal component analysis (PCA) was conducted to assess the sample distributions before and after normalization, as well as the efficacy of the ComBat function.

**TABLE 1:** Basic information on the microarray dataset.

GEO series	Number of asthmatic samples	Number of normal samples	Platform information	Attribute	Tissue
GSE41861	54	30	GPL570	Discovery set	Airway epithelium
GSE4302	42	28	GPL570	Discovery set	Airway epithelium
GSE43696	88	20	GPL6480	Test set	Airway epithelium
GSE63142	128	27	GPL6480	Test set	Airway epithelium

### 2.2 Identification of differentially expressed genes (DEGs)

The “limma” package in R software was employed to detect differentially expressed genes (DEGs) between asthma and normal tissues in the discovery set. Herein, adjusted *p* values < 0.05 and |log_2_ fold change| > 0.6 were selected as DEG-related screening criteria. Subsequently, the R packages “ggplot2” and “pheatmap” were utilized to create a volcano plot and heatmap, respectively.

### 2.3 Screening of candidate diagnostic gene as biomarker

The least absolute shrinkage and selection operator (LASSO), the support vector machine recursive feature elimination (SVM-RFE) method, and the random forest (RF) machine-learning techniques were utilized to discover new diagnostic biomarkers associated with asthma. A LASSO analysis was performed using a penalty parameter and 10-fold cross-validation through the “glmnet” package in R. The SVM-RFE technique utilizes recursion to rank features and prevent overfitting and was implemented using the “e1071” package in R. The random forest (RF) algorithm was implemented using the “randomForest” package in R to detect the point with the minimum error. A Mean Decrease in Gini score of >3 was deemed indicative of a gene strongly associated with asthma. Finally, the genes that were common to all three algorithms were chosen as the most significant genes, and their expression levels were confirmed using the test set.

### 2.4 Predictive effectiveness of biomarkers in asthma

The receiver operating characteristic (ROC) curve of the biomarker was generated using the “pROC” package in R, and the area under the curve (AUC) was computed.

### 2.5 Gene enrichment analysis

The patients with asthma were divided into two groups, low-expression and high-expression, based on the median expression of a biomarker. Subsequently, a differential expression analysis was conducted using the following criteria: |log_2_ fold change| > 0.4; adjusted *P*-value < 0.05. The relationship between the biomarker and the DEGs was examined using the “corrplot” package in R. Additionally, the “clusterProfiler” package in R was used to conduct Kyoto Encyclopedia of Genes and Genomes (KEGG) pathway analyses.

### 2.6 The sc-RNA seq data analysis

The raw data for GSE164015 and GSE193816 were obtained from the GEO database. These datasets consist of single-cell RNA sequencing data of AECs from 8 asthmatic patients. Subsequently, seurat objects were created for each sample using the Seurat package in R software. The objects were generated based on the cell-by-gene count matrix, with a minimum of 0 cells and 200 features. Accordingly, cells with nFeature_RNA >200 and nFeature_RNA <7,000, as well as a mitochondrial gene percentage <20% were selected. Raw counts were normalized using the LogNormalize method in the NormalizeData function. In addition, the top 2,000 highly variable genes (HVGs) were identified via the “FindVariableFeatures” function using the “vst” method. The data were centered and scaled using “ScaleData.” Based on these 2,000 HVGs, PCA was performed and the Harmony package was utilized to removing data-to-data batch effects. The top 50 principal components were selected for dimensionality reduction and the cells were then clustered using the “FindNeighbors” and “FindClusters” functions, with a resolution of 1.5. The cell clusters were observed using the “RunUMAP” functions. The marker genes for each cell cluster were determined using the “FindAllMarkers” function. Herein, the Cell-marker 2.0 database was utilized to categorize the cellular clusters based on their types. In addition, the analysis of intercellular communication was conducted using the “Cellchat” package, which visualized the interactions among various cell types. The “ggplot2” package was utilized to create a bubble plot illustrating the expression levels of *STEAP4* and *Macrophage migration inhibitory factor* (*MIF*) in different cell types.

### 2.7 Protein-protein docking

The target proteins’ three-dimensional structures were acquired from the PDB database. The protein structures underwent processing using PyMOL software and were subsequently uploaded to the HDOCK server for protein-protein docking. Docking scores below −200 and confidence scores above 0.7 indicate a strong probability of binding between the two proteins.

### 2.8 Establishment of ovalbumin (OVA)-induced asthma model

**FIGURE 1:**
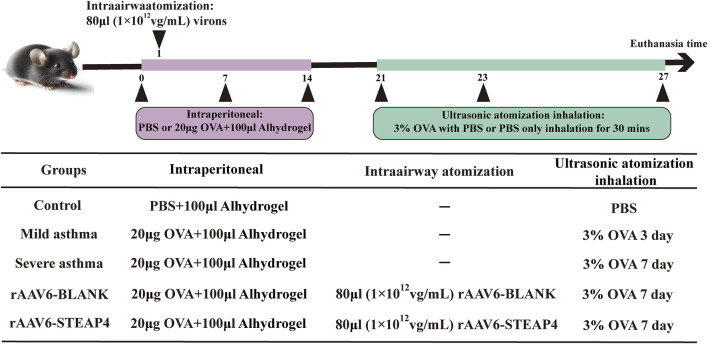
Brief protocol for the experiments and the groups used, including the control, mild asthma group, severe asthma group, rAAV6-BLANK group, and rAAV6-STEAP4 group.

The Animal Ethics and Experiments Committee of Harbin Medical University granted approval (No. 2022-DWSYLLCZ-11) for the animal studies conducted in this research. Female C57BL/6 mice, with an age of 6 weeks and a weight of approximately 20 g, were acquired from Liaoning Changsheng biotechnology Co., Ltd. The mice were housed in a Specific Pathogen Free (SPF) facility, where they were subjected to a 12-h light/dark cycle. The temperature was maintained at 22°C ± 2°C and the humidity at 50% ± 10%. The mice had unrestricted access to food and water. Before conducting the experiments, the animals were accustomed to these conditions for a period of 1 week. In the acute asthma model, mice were divided into three groups: control, mild asthma, and severe asthma, with each group containing five mice. At time points 0, 7, and 14, mice were sensitized by receiving an intraperitoneal injection of 20 μg OVA (Sigma, United States) dissolved in 100 μL PBS and combined with 100 μL aluminum hydroxide gel (InvivoGen, United States). Hanheng Biotechnology Co., Ltd. produced the recombinant adeno-associated virus type 6 (rAAV6) containing the STEAP4 cDNA sequence through synthesis. Two groups were formed: the rAAV6-BLANK group and the rAAV6-STEAP4 treatment group, each consisting of 5 mice. Within 24 h of the initial sensitization, the mice were anesthetized with 2.5% isoflurane (inhalation). Subsequently, a volume of 80 μL of STEAP4 rAAV6 (1 × 10^12^ vg/mL) was administered into the airways of mice using an intratracheal drug delivery device (Yuyan, China). This was done to induce local overexpression of STEAP4 in the AECs. As a control, the mice were injected with an empty vector that did not contain the active substance. Starting from day 21, mice in the mild asthma group were exposed to a 3% OVA solution for 30 min each day for 3 consecutive days using an ultrasonic nebulizer. Mice in the other groups were exposed to the same challenge for 7 consecutive days. After the final OVA nebulization for 24 h, mice were euthanized by cervical dislocation, and lung tissue samples were collected from the dead mice. Figure 1 depicts the chronological sequence and procedure of constructing a model.

### 2.9 Pulmonary histopathology

The lung tissues were prepared by dehydrating, embedding, and sectioning them. Then, they were stained using hematoxylin-eosin (H&E), periodic acid-schiff (PAS), and eosinophil (EOS) staining techniques. Among them, eosinophils were identified using Sirius Red stain (Solarbio, China). As mentioned earlier (Myou et al., 2003; Lu et al., 2021), HE and PAS utilize semiquantitative scores to evaluate the extent of lung inflammation and mucus secretion. Eosinophil infiltration is evaluated by calculating the proportion of eosinophils relative to the total count of nucleated cells. Two pathologists independently examined all images without knowledge of the results, with each group evaluating a minimum of 25 airways.

### 2.10 Immunohistochemistry (IHC)

The paraffin sections (5 µm) were subjected to overnight incubation at 4°C with primary antibodies against STEAP4 (Proteintech, China) and MIF (Santa Cruz, United States. On the subsequent day, detection was performed utilizing an HRP/DAB IHC detection kit (Abcam, United Kingdom). Finally, images were obtained using the Nano Zoomer S60 digital sectioning scanner (Hamamatsu Photonics K. K., Japan). The distribution area and integrated optical density (IOD) of the target protein were quantified using ImageJ (Version 1.8.0).

### 2.11 Western blot

Proteins from cell or lung tissue were extracted using RIPA buffer (Beyotime, China), and the protein concentration was determined using the bicinchoninic acid assay (BCA) approach. The proteins were transferred onto a nitrocellulose membrane (Cytiva, United Kingdom) following their separation using a 12.5% SDS-PAGE gel. The membranes were obstructed using a 5% skim milk solution and subsequently exposed to primary antibodies targeting STEAP4 (1:800) and MIF (1:600) for an entire night at a temperature of 4°C. Subsequently, the membranes were subjected to a 1-h treatment at room temperature with a secondary antibody that was labeled with horseradish peroxidase (1:5,000, Proteintech, China). The bands were visualized using an ECL reagent (Biosharp, China) and their quantities were measured using ImageJ.

### 2.12 Cell culture

The human bronchial epithelial cell line 16HBE was cultured in DMEM supplemented with 10% fetal bovine serum (Gibco, United States) at a temperature of 37°C and a CO_2_ concentration of 5%. The Th2-type inflammatory response in 16HBE cells was stimulated by the addition of house dust mite (HDM) from ALK-Abello A/S, Denmark. The MIF inhibitor ISO-1 (Sigma, United States), and the recombinant human MIF protein (rhMIF, Abcam, United Kingdom) were introduced to 16HBE in order to regulate the functioning of MIF signaling.

### 2.13 Plasmid transfection

Inovogen Biotechnology Co., Ltd. inserted the human STEAP4 cDNA into the pLV-puro vector through the process of cloning. The overexpression experiment involved transfecting 293 T cells with either the control vector pLV-puro or the overexpression vector pLV-puro-hSTEAP4, along with lentiviral packaging plasmids, following the instructions provided by the manufacturer. After 48 h, the supernatant containing the genetically modified lentivirus was gathered and subsequently utilized to infect 16HBE cells. The siRNA targeting the *STEAP4* gene (GenePharma, China) or a control siRNA with no specific target was introduced into 16HBE cells using Lipofectamine 2000 (Thermo Fisher Scientific, United States) for the purpose of conducting knockdown experiments. The concentration of plasmids used for cell transfection is between 0.7 and 1 μg/μL.

### 2.14 Immunofluorescence (IF)

The cells were treated with a 4% paraformaldehyde solution and allowed to fix for a duration of 15 min. Following a 15-min incubation in QuickBlock buffer (Beyotime, China), the cells were subjected to overnight incubation at 4°C with primary antibodies targeting STEAP4 and MIF. Subsequently, the cells were cultured with secondary antibodies labeled with CoraLite488 and Rhodamine (1:100, Proteintech, China) at room temperature for a duration of 2 h. Following the application of DAPI stain, the cells were examined using a laser confocal microscope (Olympus, Japan).

### 2.15 Real-time quantitative reverse transcription–polymerase chain receptor (qRT–PCR)

The extraction of total RNA from cells or lung tissue was performed using TRIzol reagent (Invitrogen, United States). The Nanodrop 2000 spectrophotometer (Thermo Fisher Scientific, United States) was utilized to measure the purity and concentration of RNA samples. The High Capacity cDNA Reverse Transcription Kit (Thermo Fisher Scientific, United States) was used to reverse transcribe 1 μg of RNA from each sample. Subsequently, qRT-PCR was performed using PowerUp SYBR Green Master Mix (Thermo Fisher Scientific, United States) and a fluorescence quantitative PCR instrument (Roche, Switzerland). The primers were designed using Prism 6.0 software and synthesized by Shanghai Sangon Biotech Co., Ltd.

### 2.16 Determination of cytokines

The levels of Th2 cytokines (IL-4, IL-25, IL-33) in the bronchoalveolar lavage fluid (BALF) of mice and the culture supernatant of 16HBE cells were measured using the ELISA method. The measurement of the aforementioned cytokines was carried out according to the experimental procedures provided by the manufacturer (Hangzhou Lianke tech. Co. Ltd., China) in their ELISA kits.

### 2.17 Statistical analysis

The experimental data was presented in the form of the mean ± standard deviations (SDs). Statistical plotting was performed using GraphPad Prism 9.5 software. In addition, the comparisons between sample groups were carried out using a two-tailed Student’s t-test. A P-value less than 0.05 was considered to be statistically significant (^*^ *P*-value < 0.05; ^**^ *P*-value < 0.01; ^***^ *P*-value < 0.001).

## 3 Results

### 3.1 Data preprocessing and identification of DEGs

**FIGURE 2:**
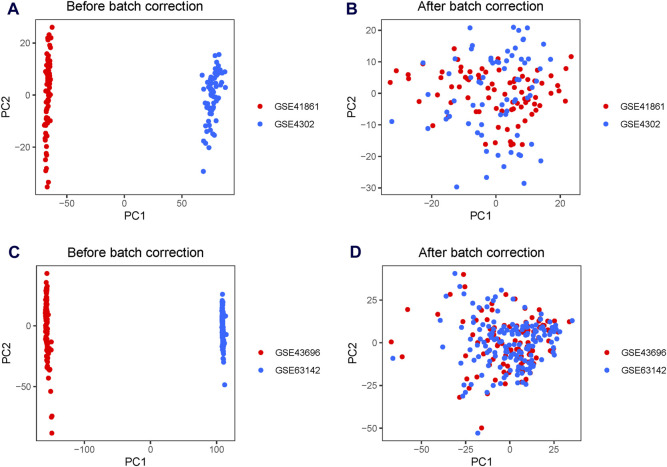
Principal component analysis (PCA) plots for the merged gene expression matrix before and after eliminating the batch effect. PCA plots for the discovery set **(A)** before and **(B)** after normalization using the Surrogate Variable Analysis (SVA) package. PCA plots for the test set **(C)** before and **(D)** after normalization using the SVA package. The points represent each sample.

**FIGURE 3:**
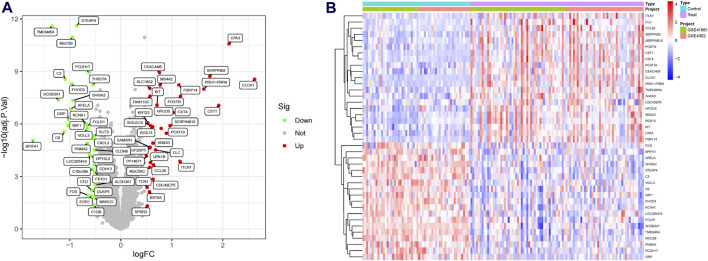
Differentially expressed genes (DEGs) in asthmatic and normal samples. **(A)** Volcano plot for the DEGs. **(B)** Heatmap for the DEGs.

A significant batch effect was evident across the various datasets (Figures 2A,C). The “SVA” package in the R software was used to normalize the merged gene expression matrix, and the effective elimination of the batch effects in the discovery and test sets was revealed by a PCA plot (Figures 2B,D). A total of 40 DEGs were identified in the discovery set, with 21 genes showing upregulation and 19 genes showing downregulation. The volcano map shown in Figure 3A illustrates the spatial distribution of the DEGs, whereas the heatmap in Figure 3B displays the expression levels of the DEGs in each sample.

### 3.2 Screening and validation of the diagnostic biomarkers

**FIGURE 4:**
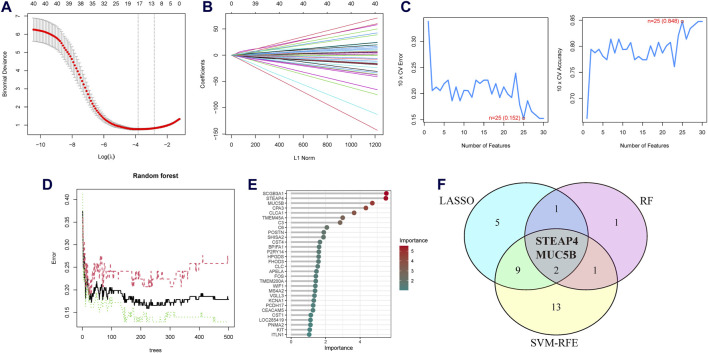
Screening process for the candidate asthma biomarkers. **(A)** Cross-validation plot of the least absolute shrinkage and selection operator (LASSO) model. The horizontal axis represents the logarithmic values of the regularization parameter (λ), while the vertical axis represents the cross-validation error. The curve shows the performance of the model at different λ values. The vertical dashed line represents the λ value corresponding to the minimum cross-validation error, which is the optimal λ value. **(B)** Coefficient path plot of the LASSO model. The horizontal axis represents the L1 norm, while the vertical axis represents the feature coefficients of each gene. Each line represents the trajectory of feature coefficient changes for a gene at different λ values. **(C)** Performance plots of the support vector machine recursive feature elimination (SVM-RFE) algorithm in the screening biomarkers. It shows the relationship between the change in the number of features and the model’s accuracy and error rate. The closer the accuracy is to 1 and the error rate is to 0, the better the performance of the model, as well as the number of selected biomarkers. **(D)** The relationship plot between the number of trees and the error rate in the random forest (RF) algorithm. **(E)** Ranking of genes based on their Mean Decrease in Gini coefficients. **(F)** Venn diagram of the intersecting characteristic genes mined by the LASSO, SVM-RFE, and RF algorithms.

**FIGURE 5:**
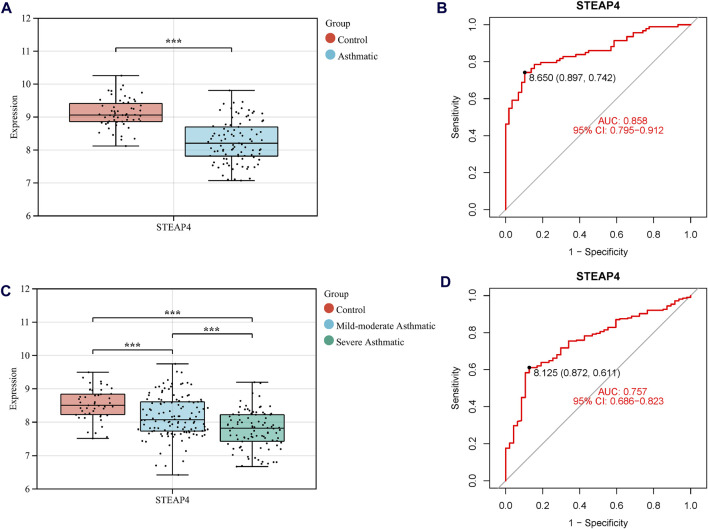
The mRNA level of *STEAP4* and its diagnostic effects. Box diagram of the mRNA levels of *STEAP4* in **(A)** the discovery set and **(C)** the test set. Receiver operating characteristic (ROC) curves of *STEAP4* in the **(B)** discovery set and **(D)** test set. ^***^ *P*-value < 0.001.

Three machine learning algorithms were utilized to identify significant DEGs that can serve as biomarkers for asthma. Out of these DEGs, 17 were identified using the LASSO regression method (Figures 4A,B). In addition, the SVM-RFE algorithm was used to extract twenty-five characteristic genes from the DEGs (Figure 4C). The RF algorithm successfully identified five genes (Figures 4D,E). STEAP4, the overlapping key gene of the three algorithms, was selected as the potential biomarker (Figure 4F). Compared to healthy individuals, the level of *STEAP4* mRNA was markedly decreased in asthmatic patients, (Figure 5A). Furthermore, in the test set, the expression level of the *STEAP4* gene decreased in proportion to the severity of asthma, providing additional confirmation of the precision and consistency of this biomarker (Figure 5C). In addition, the AUC value of *STEAP4* was 0.858 in the discovery set and 0.757 in the test set (Figures 5B,D), demonstrating that *STEAP4* exhibited significant diagnostic utility in distinguishing between the asthma group and the normal group.

### 3.3 Functional analysis of STEAP4-related DEGs

**FIGURE 6:**
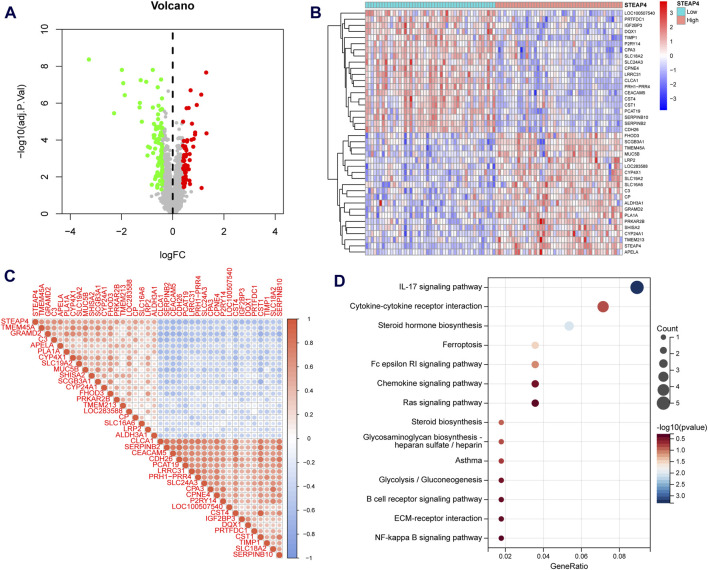
Functional enrichment analysis of the differentially expressed genes (DEGs) between the *STEAP4*-high and *STEAP4*-low groups of patients with asthma. **(A)** Volcano plot for the DEGs. **(B)** Heatmap for the DEGs. **(C)** Correlation between *STEAP4* and the DEGs. **(D)** Bubble plots for the Kyoto encyclopedia of genes and genomes (KEGG) pathway enrichment of the DEGs.

Asthmatic patients were classified into high-*STEAP4* and low-*STEAP4* groups based on the median mRNA level of *STEAP4*. The volcano map (Figure 6A) demonstrates that there were a total of 146 DEGs between the two subgroups. Among these DEGs, 61 genes were upregulated and 85 genes were downregulated. The heatmap exhibits the top 40 DEGs determined by the |log_2_ fold change| value (Figure 6B). In addition, the correlation heatmap indicates a significant positive correlation between *STEAP4* and genes such as *CP, LRP2*, and *SHISA2*, as well as a significant negative correlation between *STEAP4* and genes such as *CST1, CLCA1,* and *CPA3* (Figure 6C).

An enrichment analysis of 146 DEGs was conducted to gain insight into the biological function of *STEAP4*. The KEGG analysis revealed that the DEGs primarily regulated the interleukin (IL)-17 signaling pathway, steroid hormone biosynthesis, cytokine-cytokine receptor interaction, Fc epsilon RI signaling pathway and ferroptosis, all of which are closely related to asthma (Figure 6D).

### 3.4 Intercellular communication influenced by STEAP4

**FIGURE 7:**
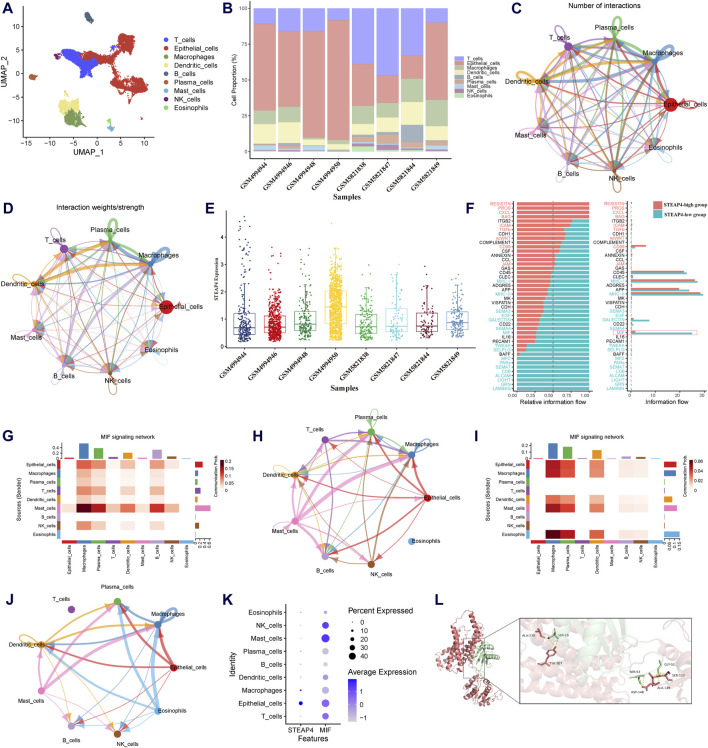
Exploration of the downstream signaling pathways of STEAP4 utilizing the scRNA-seq data. **(A)** Uniform manifold approximation and projection (UMAP) plot of the scRNA-seq data annotated with 7 distinct cell types. **(B)** Proportion of the cell types in the tracheal brush samples from eight asthmatic patients. **(C)** Interaction net count plot of 7 cell types. The size of the colored circles is proportional to the number of cells. Cells emitting arrows express ligands; the arrows point towards cells that express receptors. The thickness of the lines is proportional to the number of ligand-receptor pairs. **(D)** The interaction net weight plot of 7 cell types. The thickness of the line is proportional to the strength of the ligand-receptor pair interaction. **(E)** Expression of *STEAP4* in each sample of the scRNA-seq data. **(F)** All significant signaling pathways were ranked based on their differences in the overall information flow within the inferred networks between the *STEAP4* low expression samples and the *STEAP4* high expression samples. The blue signaling pathways were more enriched in the *STEAP4* low expression samples, and the red signaling pathways were more enriched in the *STEAP4* high expression samples. **(G)** The intercellular communication probability between different cell types in the *MIF* signaling network in the high *STEAP4* expression group. **(H)** MIF signaling pathway network in the high *STEAP4* expression group. **(I)** The intercellular communication probability between different cell types in the *MIF* signaling network in the low *STEAP4* expression group. **(J)** *MIF* signaling pathway network in the low *STEAP4* expression group. **(K)** Dot plot depicting percent expression and average expression of STEAP4 and MIF across different cell types. **(L)** Visualization of the docking between STEAP4 and MIF proteins. The red structure represents the STEAP4 protein, the green structure represents the MIF protein, and the yellow dashed lines between them indicate hydrogen bond.

To determine influenced of *STEAP4* in intercellular communication, the scRNA-seq data obtained from tracheal brush samples collected from 8 patients diagnosed with asthma were processed and screened. By employing graph-based clustering of uniform manifold approximation and projection (UMAP), a total of 24,202 cells were segregated into 34 distinct clusters. These clusters were subsequently categorized into 9 different cell types, viz. T cells, B cells, epithelial cells, macrophages, mast cells, DCs, plasma cells, eosinophils and natural killer cells (Figure 7A). The proportions of different cell types in tracheal brush samples collected from 8 patients with asthma are shown in Figure 7B.

We utilized Cellchat to investigate the quantity and strength of interactions between ligand-receptor pairs across seven distinct cell types in the dataset. This analysis revealed a wide-ranging and dynamic exchange of information among cells (Figures 7C,D). Subsequently, the expression levels of *STEAP4* in each sample were obtained (Figure 7E). Based on the expression levels of *STEAP4*, the scRNA-seq samples were classified into a high expression group (GSM4994950, GSM4994948, GSM5821849) and a low expression group (GSM4994944, GSM4994946, GSM5821838). Accordingly, the cellular signaling between the *STEAP4* low expression group and the *STEAP4* high expression group was compared. The analysis revealed that the MIF signaling pathway was more prevalent in the *STEAP4* low expression group (Figure 7F). Figures 7H,J illustrate the MIF signaling pathway network in the two groups, respectively. On the other hand, Figures 7G,I provide a detailed illustration of the communication probability among various cells in this specific signaling pathway. By analyzing the expression of *STEAP4* and *MIF* in different cell types, it was discovered that both *STEAP4* and *MIF* are predominantly expressed in epithelial cells (Figure 7K). Accordingly, it was hypothesized that *STEAP4* participates in the regulation of the Th2-type inflammatory response in AECs by influencing the *MIF* signaling pathway. However, this hypothesis needs to be verified through additional experiments.

The protein-protein docking analysis reveals that the docking score between STEAP4 and MIF is −363.68, accompanied by a confidence score of 0.9863. This outcome strongly indicates that they have the ability to spontaneously create a stable complex. Figure 7L illustrates the specific information regarding the molecular interaction between STEAP4 and MIF. This finding offers compelling molecular-level evidence of the interaction between STEAP4 and MIF.

### 3.5 Expression of STEAP4 and MIF in AECs of allergic asthma mice

**FIGURE 8:**
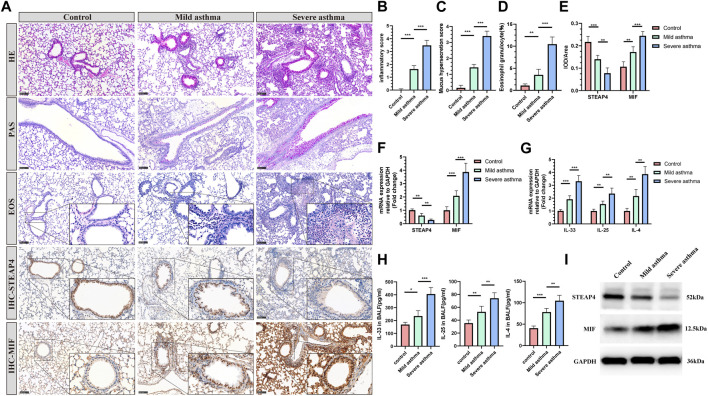
Levels of STEAP4 and MIF proteins and mRNA in the AECs of allergic asthma mice. **(A)** Representative results for, hematoxylin and eosin (H&E), periodic acid-schiff (PAS), eosinophil (EOS) staining, and immunohistochemistry (IHC) in the lung tissue samples of the control, mild asthma, and severe asthma groups. The scale bar is set at 100 μm. The dashed box represents the area magnified. **(B)** Quantification of the H&E results. **(C)** Quantification of the PAS results. **(D)** Quantification of the EOS results. **(E)** Quantification of the STEAP4 and MIF IHC results. **(F)** *STEAP4* and *MIF* mRNA levels were analyzed in the lung tissues of three groups of mice using RT-qPCR. **(G)** *IL-33, IL-25,* and *IL-4* mRNA levels were analyzed in the lung tissues of three groups of mice using RT-qPCR. **(H)** Determination of IL-25, IL-33, IL-4 cytokine levels in BALF of three groups of mice by ELISA. **(I)** STEAP4 and MIF protein levels were analyzed in the lungs of the three groups of mice using Western blot. All results were from 5 mice. ^*^ *P*-value < 0.05; ^**^ *P*-value < 0.01; ^***^ *P*-value < 0.001.

The establishment of models for mild and severe asthma in mice was achieved by simulating different levels of asthma severity using OVA nebulization for varying durations. With an increase in the duration of OVA nebulization, there is a clear rise in the presence of inflammatory cells surrounding the airways, the production of mucus in the airways, and the proportion of eosinophils (Figures 8A–D). Additionally, the RT-qPCR assays demonstrated a significant increase in the expression levels of *IL-33*, *IL-25*, and *IL-4* cytokines (Figure 8G). And the decreased levels of IL-33, IL-25, and IL-4 in BALF of mice are measured by ELISA (Figure 8H). As observed, the Th2 inflammatory response in asthmatic mice led to a gradual decrease in the levels of STEAP4 protein and mRNA in airway epithelial cells, which was negatively correlated with the severity of asthma. Conversely, the protein and mRNA expression levels of MIF gradually increased, demonstrating a positive correlation with the severity of asthma (Figures 8A,E,F,I).

### 3.6 The effect of overexpression of STEAP4 in mouse AECs on the MIF signaling pathway and Th2 inflammation

**FIGURE 9:**
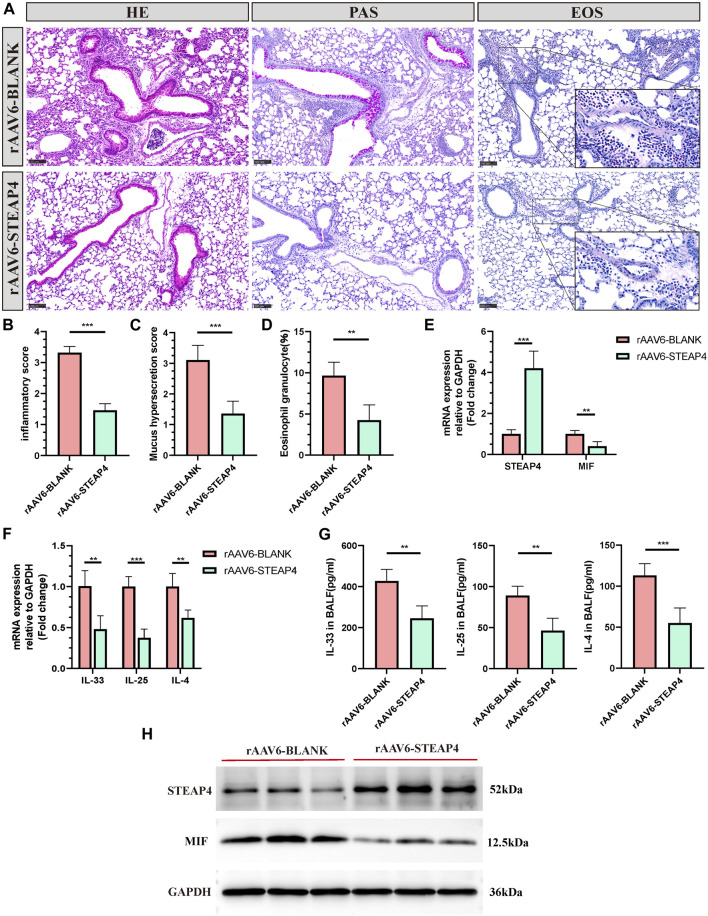
Effects of the overexpression of STEAP4 on MIF expression levels in the AECs of allergic asthma mice. **(A)** Representative staining results of hematoxylin and eosin (H&E), periodic acid-schiff (PAS), and eosinophil (EOS) staining in the lung tissue samples of asthmatic mice treated with rAAV6-blank/STEAP4 and then induced by OVA for 7 days. **(B)** Quantification of the HE results. **(C)** Quantification of the PAS results. **(D)** Quantification of the EOS results. **(E)** The mRNA levels of STEAP4 and MIF were analyzed in the lung tissues of mice from the rAAV6-blank, and rAAV6-STEAP4 groups using RT-qPCR. **(F)** *IL-33*, *IL-25*, and *IL-4* mRNA levels were analyzed in the lung tissues of the rAAV6-blank and rAAV6-STEAP4 groups using RT-qPCR. **(G)** Determination of IL-25, IL-33, IL-4 cytokine levels in BALF of two groups of mice by ELISA. **(H)** STEAP4 and MIF protein levels were analyzed in the lungs of two groups of mice using Western blot. All results were from 5 mice. ^**^ *P*-value < 0.01; ^***^ *P*-value < 0.001.

In order to investigate the potential connection between STEAP4 levels and the development of allergic asthma and MIF levels, rAAV6-STEAP4 was injected into the airways of sensitized mice using a pulmonary administration device. This was done to induce an overexpression of STEAP4. Subsequently, the mice were exposed to continuous 3% OVA nebulization for a period of 7 days. The levels of STEAP4 protein and mRNA were increased in mouse lung tissue treated with rAAV6-STEAP4 compared to wild-type severe asthmatic mice and the rAAV6-blank group. This suggests that asthma mice with AECs overexpressing STEAP4 were successfully created (Figures 9E,H). In addition, the overexpression of STEAP4 in AECs resulted in notable reductions in the infiltration of inflammatory cells, secretion of mucus, and the proportion of eosinophils (Figures 9A–D). Moreover, the expression levels of *IL-33*, *IL-25*, and *IL-4* cytokines in lung tissue and BALF were decreased in the rAAV6-STEAP4 group compared to the rAAV6-blank group (Figures 9F,G). Importantly, the expression levels of MIF protein and mRNA in AECs of the rAAV6-STEAP4 group were significantly reduced (Figures 9E,H). These findings provide evidence that STEAP4 has the ability to suppress the MIF signaling pathway and Th2-type inflammatory responses in AECs, thus supporting the initial hypothesis.

### 3.7 The effects of overexpression and knockdown of STEAP4 on the MIF signaling pathway in 16HBE cells

**FIGURE 10:**
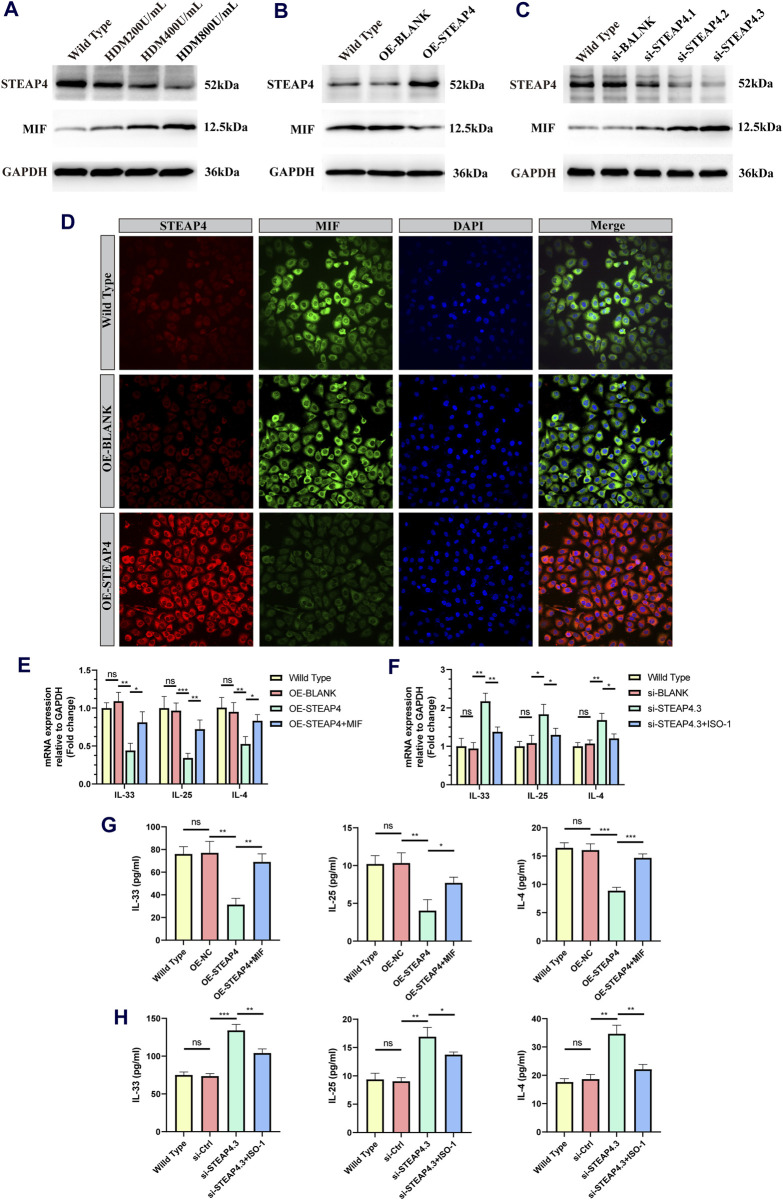
Effects of the STEAP4 protein expression on MIF as well as cytokines expression levels in 16HBE cells. **(A)** 16HBE cells were stimulated with house dust mite (HDM), and the protein levels of STEAP4 and MIF were detected using Western blotting. **(B)** The expression levels of STEAP4 and MIF in 16HBE cells that were transfected with the recombinant plasmid pLV-hSTEAP4-puro and control empty vector were detected by Western blot. **(C)** After transfecting 16HBE cells with siRNA targeting STEAP4, the expression levels of STEAP4 and MIF proteins were detected using Western blot. **(D)** IF staining of STEAP4 and MIF in 16HBE cells. **(E)** Effects of overexpressing STEAP4 on *IL*-33, *IL*-25, and *IL*-*4* mRNA levels in 16HBE cells. **(F)** Effects of knocking down STEAP4 on *IL-33*, *IL-25*, and *IL-4* mRNA levels in 16HBE cells. **(G)** Effect of overexpression of STEAP4 on the levels of IL-33, IL-25 and IL-4 cytokines in supernatant of 16HBE. **(H)** Effects of knocking down STEAP4 on IL-33, IL-25, and IL-4 cytokines levels in supernatant of 16HBE. All results were repeated three times. ^*^ *P*-value < 0.05; ^**^ *P*-value < 0.01; ^***^ *P*-value < 0.001.

In order to confirm the functionality of AECs, *in vitro* experiments were conducted, taking into account that the use of HDM can more closely simulate the allergic reactions experienced by human AECs in real-life settings. Accordingly, three different concentrations of HDM (200, 400, and 800 U/mL) were employed to stimulate 16HBE cells. After 24 h, the protein levels of STEAP4 and MIF were evaluated using Western blot analysis. The results suggest that as the dosage of HDM increased, the expression level of STEAP4 decreased gradually decreased, while the expression level of MIF increased gradually. These findings are in line with previous *in vivo* experimental results (Figure 10A).

Subsequently, by employing the liposome transfection technique, 16HBE cell models that exhibit both overexpression and knockdown of STEAP4 were effectively developed. The cells in the OE-STEAP4 group showed a significant increase in the expression of STEAP4 protein, while the expression of MIF significantly decreased (Figure 10B). Following the transfection of three distinct siRNA targets into 16HBE cells, the expression of STEAP4 was significantly reduced in the siSTEAP4.1, siSTEAP4.2, and siSTEAP4.3 groups, with the most pronounced reduction observed in the siSTEAP4.3 group. As the expression of STEAP4 decreased, there was a gradual increase in the expression of MIF (Figure 10C). Moreover, the outcomes of IF staining were in agreement with the findings of the Western blot analysis. In addition, the proteins STEAP4 and MIF were observed in both the cytoplasm and membrane of the cells, demonstrating a co-localization phenomenon (Figure 10D). These findings provide additional evidence that STEAP4 may impact the biological functions of AECs by decreasing the expression of MIF within the cytoplasm.

### 3.8 STEAP4 reduces cytokine release by blocking the MIF signaling pathway

A concentration of 400 U/mL of HDM was introduced to 16HBE cells that were either overexpressing or knocking down STEAP4, and the levels of *IL-25, IL-33,* and *IL-4* cytokines were measured using RT-qPCR and ELISA after 24 h. The experimental results suggest that the levels of *IL-25, IL-33*, and *IL-4* were lower in OE-STEAP4 cells as well as in the supernatant compared to wild-type and OE-BLANK cells. The exogenous addition of rhMIF was able to reverse the decrease in cytokine levels induced by the overexpression of STEAP4 (Figures 10E,G). The levels of *IL-33, IL-25,* and *IL-4* were significantly increased in si-STEAP4.3 cells as well as in the supernatantcompared to wild-type and si-blank cells. This increase in cytokine levels was reversed when the MIF signaling inhibitor ISO-1 was introduced (Figures 10F,H). These results demonstrate how STEAP4 inhibits the production of IL-25, IL-33, and IL-4 cytokines in AECs by obstructing the MIF signaling pathway, consequently impacting the Th2 inflammatory response.

## 4 Discussion

By employing a combination of three different machine learning algorithms, two key DEGs between asthmatic patients and the control group, *viz*. STEAP4 and MUC5B, were successfully identified from the microarray data of AECs. Given that the role of MUC5B in the pathogenesis of asthma has been extensively studied, this particular gene was not addressed in the present study (Lachowicz-Scroggins et al., 2016; Bonser and Erle, 2017). STEAP4 belongs to the metalloreductase family and plays a crucial role in the reduction and transport of iron and copper (Scarl et al., 2017). In addition, it is involved in mitochondria electron transport, anti-inflammatory processes, and glucose metabolism (Chen et al., 2014). Within the synovium of individuals with rheumatoid arthritis, STEAP4 inhibits inflammation by blocking the activity of inflammatory cytokines (specifically IL-6 and IL-8), impeding cell proliferation, and inducing apoptosis of fibroblast-like synoviocytes (Tanaka et al., 2012). Within adipocytes, STEAP4 reduces the levels of pro-inflammatory cytokines (MCP-1, IL-6, and TNF-α), while simultaneously increasing the levels of anti-inflammatory cytokines (IL-10). This leads to a reduction in the infiltration of macrophages and an improvement in insulin sensitivity (Han et al., 2013). To date, studies that have focused on the role of STEAP4 in AECs, the role of STEAP4 in AECs have been largely limited. As a result, the present study aims to address this knowledge gap by examining the function of STEAP4 in AECs.

Herein, the expression of STEAP4 was found to be decreased in the AECs of asthmatic patients for the first time. Furthermore, this reduction in expression was found to be negatively associated with the severity of the disease. In addition, the AUC of STEAP4 was greater than 0.75 in both the discovery and test sets, suggesting that it possesses a substantial diagnostic value. Moreover, the expression of STEAP4 in AECs demonstrated a positive correlation with genes such as CP, LRP2, and SHISA2, and a negative correlation with genes such as CST1, CLCA1, and CPA3. All of these genes were associated with the pathogenesis of asthma. For instance, CST1 promotes airway eosinophilic inflammation by activating the AKT signaling pathway (Du et al., 2023). On the other hand, the expression of CLCA1 was found to have a positive correlation with the levels of serum IL-13 in children diagnosed with asthma (Xu et al., 2022). Moreover, the expression of CPA3 in the sputum cells of asthmatic patients was found to be increased, and it has been used as a reliable indicator for predicting responsiveness to corticosteroids as well as the occurrence of future exacerbations (Baines et al., 2014; Berthon et al., 2017; Fricker et al., 2019). In addition, the KEGG enrichment analysis suggests that STEAP4 may play a crucial role in regulating IL-17 signaling, steroid hormone biosynthesis, and ferroptosis. IL-17 is the classical cytokine that is secreted by Th17 cells (Camargo et al., 2023). Herein, asthmatic patients exhibited markedly elevated IL-17 levels in bronchial biopsies, sputum, BALF and sera, in comparison to control subjects. These elevated levels of IL-17 were found to be directly associated with the severity of the disease (Silverpil and Lindén, 2012; Chesné et al., 2014). Moreover, the expression of IL-17 in the airways was found to contribute to the advancement of asthma by inducing the infiltration of neutrophils, excessive production of mucus, an increase in the number of goblet cells, the differentiation of myofibroblasts, and the proliferation of airway smooth muscles (Newcomb and Peebles, 2013; Ramakrishnan et al., 2019). Ferroptosis, a non-apoptosis regulated cell death, is typically characterized by iron-dependent lipid peroxidation and reactive oxygen species (ROS) accumulation (Stockwell et al., 2017). Herein, ferroptosis was induced in the lung tissue of the asthmatic mouse model through exposure to HDM. Subsequent treatment with ferroptosis inhibitors was found to alleviate airway inflammation and reduce the death of AECs, indicating a strong association between ferroptosis in AECs and asthma (Tang et al., 2021; Zeng et al., 2022). These findings indicate that STEAP4 has the potential to serve as a biomarker for asthma. To further confirm the accuracy of the previous findings, animal models using both mild and severe asthma mice were established, and a Th2 inflammation model of 16HBE cells was generated. The findings demonstrated a decrease in the expression level of STEAP4, which negatively correlated with the degree of Th2 inflammation and was consistent with the results obtained from bioinformatics-based investigations conducted previously.

ScRNA-seq offers a distinct advantage over bulk RNA sequencing by allowing the detection of variations in RNA expression among various cell types. Accordingly, this enables the investigation of cellular signaling at the level of individual cells. Herein, the intercellular communication in scRNA-seq data between samples with high and low expression of *STEAP4* was conducted. The analysis revealed that the *MIF* pathway was found to be upregulated in samples with low expression of *STEAP4*. MIF was originally characterized as a T lymphocyte–derived protein that has the ability to inhibit the random migration of macrophages and a key modulator of both the inflammatory and immune responses. (Hoi et al., 2007). Existing studies have shown that the levels of MIF in BALF, induced sputum, and serum were found to be significantly elevated in individuals with asthma compared to the control group. Furthermore, these levels were found to be positively associated with the severity of asthma (Yamaguchi et al., 2000; Florez-Sampedro et al., 2020). Conversely, upon MIF deficiency or inhibition, asthmatic features, including eosinophil and neutrophil counts, airway hyperresponsiveness, airway smooth muscle thickness, levels of Th2 cytokines, and IgE titers, were found to be significantly reduced (Mizue et al., 2005; Kobayashi et al., 2006; Chen et al., 2010; Bozza et al., 2020; Lan et al., 2020). MIF also plays a role in mediating resistance to the anti-inflammatory effects of steroids (Calandra and Bucala, 2017). Accordingly, STEAP4 was postulated to be involved in regulating Th2-type inflammatory responses by inhibiting MIF function. Consequently, STEAP4 was upregulated in the AECs of asthmatic mice via rAAV6. The rAAV6-STEAP4 group of mice exhibited a notable reduction in Th2-type inflammatory responses in lung tissues and the expression level of MIF in AECs, when compared to the control group, thus validating the aforementioned hypothesis.

Nonetheless, the rAAV6 vector is not specifically engineered for infecting AECs. Furthermore, the airway environment contains a large number of inflammatory cells. Thus, the present study constructed STEAP4-overexpressed and knocked-down 16HBE cell lines to verify the independent functions of AECs. When 16HBE overexpressed STEAP4, the expression of MIF decreased significantly. Conversely, when STEAP4 was knocked down in 16HBE cells, the expression of MIF increased. Lan et al. found that elevating the MIF expression promoted IL-4, IL-5, IL-13 secretion by 16HBE (Lan et al., 2020). García-Arellano et al. have demonstrated that MIF can promote the secretion of IL-25, IL-31, and IL-33 b y peripheral blood mononuclear cells in individuals diagnosed with rheumatoid arthritis (García-Arellano et al., 2021). In this context, IL-25 and IL-33, released as alarmins by AECs in asthmatic patients, have a crucial function in promoting Th2-type inflammation (Duchesne et al., 2022). Nevertheless, there is currently a lack of studies that have reported on whether MIF stimulates the release of IL-25 and IL-33 in AECs. Therefore, additional investigations were conducted to address this issue. The findings indicated that the levels of IL-33, IL-25, and IL-4 released by 16HBE cells overexpressing STEAP4 were significantly lower compared to the control group. However, the addition of rhMIF successfully restored the levels of these cytokines. Conversely, the depletion of STEAP4 in 16HBE cells resulted in a notable increase in the release of these cytokines. However, when the MIF inhibitor ISO-1 was added, the levels of cytokines were reversed. Thus, STEAP4 was observed to potentially decrease the release of IL-33, IL-25, and IL-4 by suppressing the MIF signaling pathway. These findings offer fresh theoretical backing for considering STEAP4 as a promising target for treating asthma.

Although the aforementioned results are promising, it is crucial to recognize their limitations. First, the clinical data for this study were acquired from public databases, and the samples were deficient in clinical information, rendering it impossible to establish the correlation between STEAP4 and clinicopathological aspects. Secondly, the outcomes of the study may have been influenced by the small sample size, which could have introduced bias. Thus, to ensure the reliability, reproducibility, and applicability of these findings, it is necessary to validate them in future clinical samples. Finally, this work conducted bioinformatic analyses with scRNA-seq and array data from tracheal brush samples. These samples mainly collect airway epithelial cells and immune cells, and might be inadequate to represent those cells from deeper tissues or different regions (such as fibroblasts, smooth muscle cells, and vascular cells). Therefore, we will further investigate the expression of STEAP4 in other cell types through airway biopsies in our subsequent studies. These factors warrant future research.

## 5 Conclusion

STEAP4 was found to have the potential to serve as a biomarker and be an effective therapeutic target for asthma. The expression level of STEAP4 was confirmed to be reduced in the AECs of individuals with asthma and was inversely associated with the severity of the condition. Additionally, STEAP4 was found to inhibit the release of cytokines IL-33, IL-25, and IL-4 by obstructing the MIF signaling pathway, consequently suppressing Th2-type inflammatory reactions.

## Data Availability

The datasets presented in this study can be found in online repositories. The names of the repository/repositories and accession number(s) can be found below: Gene Expression Omnibus (GEO), accession number GSE41861, GSE4302, GSE43696, GSE63142, GSE164015 and GSE193816.
